# Evaluating the role of biotic and abiotic factors in hardwood tree development in the Central Hardwood Region (CHR)

**DOI:** 10.1186/s40793-026-00935-2

**Published:** 2026-07-14

**Authors:** Shaneka S. Lawson, Juan P. Frene, Niall D. Lue Sue

**Affiliations:** 1 USDA Forest Service, Hardwood Tree Improvement and Regeneration Center (HTIRC), Department of Forestry and Natural Resources, Northern Research Station, Purdue University, 715 West State Street, West Lafayette, IN PFEN226C, 47907 USA; 2 School of Biosciences, University of Nottingham, Sutton Bonington, Nottingham, LE12 5RD UK; 3 Present Address: Agricultural Science Center, New Mexico State University, Clovis, NM 88101 USA

**Keywords:** Hardwood trees, *Quercus rubra*, *Juglans nigra*, Plant growth, Bacteria

## Abstract

**Background:**

Hardwood tree growth and development are influenced by the interplay between soil microbiota and tree physiology. Though unclear mechanistically, microbial community composition is associated with tree growth. We investigated how soil chemistry and prokaryotic communities impacted *Quercus rubra* (red oak) and *Juglans nigra* (black walnut) growth across three plantations in Indiana and Michigan. Trees exhibiting above- (high-performing or healthy) or below-average (low-performing or poor) growth rates were compared. Correlations with soil properties and microbial composition (16 S rRNA sequencing) were assessed to identify factors associated with variations in mean dominant tree height and diameter at breast height (DBH).

**Results:**

Tree growth differed by site, species, and performance class. High-performing trees showed 25–40% higher AGR and 10–20% higher RGR across study sites. Larger trees occurred in Southern and Western Indiana than in Michigan. *J. nigra* outperformed *Q. rubra* in Indiana, and the reverse occurred in Michigan. DH and DBH were strongly correlated (R^2^ = 0.8046, *P* < 0.001), and soil pH differed by species. Organic C, N, and CEC differed among species, but not among sites or performance class. Prokaryotic composition varied by species and site and explained 10.7%, 9.4%, and 7.6% of the variance. Microbial community structure was linked to DBH in *J. nigra* (*r* = 0.14, *P* = 0.002) and *Q. rubra* (*r* = 0.078, *P* = 0.028), and height only in *J. nigra* (*r* = 0.20, *P* = 0.001). Differential abundance analysis identified 88 and 122 growth-associated ASVs in *J. nigra* and *Q. rubra*, respectively. Random Forest models explained that soil texture (sand and silt) and microbial taxa (*Bacillus*, *Arthrobacter*, GP1, GP3) are key elements for growth.

**Conclusions:**

Hardwood growth is more strongly associated with soil texture and specific microbial taxa than with overall microbial diversity. These species-dependent relationships suggest that microbial indicators of growth performance differ among hardwood species. Overall, the results improve our understanding of plant, soil, and microbe interactions and support using microbial indicators to monitor and improve hardwood plantation performance. Identifying microbial indicators associated with superior growth could inform reforestation and silvicultural practices in temperate hardwood ecosystems.

**Supplementary Information:**

The online version contains supplementary material available at https://doi.org/10.1186/s40793-026-00935-2.

## Introduction

Natural and managed forests support biodiversity, forest management, and research by providing essential native habitats and contributing to regional and national economies. Growth and development of hardwood trees (e.g., chestnut, oak, maple, hickory) depend strongly on soil composition, roots, and the surrounding microbial community [1]. Plants, soil, and microbes collectively control growth, nutrient cycling, and ecosystem health, with long-term forest sustainability depending on these belowground processes. The functioning and long-term sustainability of forest ecosystems are strongly mediated by belowground processes [2]. Understanding these complex mechanisms is essential for maintaining healthy forests and plantations [3, 4].

Soil microbiota facilitate plant nutrient uptake, organic matter decomposition, and release of several primary macronutrients vital for growth and reproduction, like nitrogen (N), phosphorus (P), and potassium (K). Tree species occupy distinct ecological niches and can differ in the composition of their associated soil microbial communities. *Quercus rubra* (Northern red oak, *Q. rubra*) and *Juglans nigra* (black walnut, *J. nigra*) are widespread and highly valued species in forests and plantations throughout the central hardwood region of the United States [5]. They provide an ideal system for examining species-specific relationships among tree growth, soil properties, and microbial communities. The two species differ in soil adaptation, with *J. nigra* generally preferring moderately-textured soils such as sand-loam or loam. *Q. rubra* is less particular, thrives across a broader range of acidic soils, and has lower N, P, and K requirements [6]. These contrasting conditions create distinct belowground environments that may support varying microbial communities and interaction networks.

Soil moisture is a key regulator of microbial activity and impacts soil carbon (C) release into the atmosphere. Although higher soil moisture typically stimulates microbial activity, multiple interacting factors, including historical climate, site history, management practices, and edaphic and biotic factors, may influence present-day microbial responses and contribute to regional variability in hardwood tree establishment and growth [7]. Relationships between microbes, plants, and soil are inherently bidirectional. The root exudates, litter inputs, and nutrient uptake from trees influence microbial community composition just as microbes can influence tree growth and performance. Therefore, observed variations in tree growth may reflect feedback among the many plant-driven and microbially-mediated processes as opposed to being driven unidirectionally. Specific soil bacteria can also influence tree height and diameter by affecting nutrient availability, phytohormone regulation, disease resistance, and soil-water relations [7]. Common bacterial phyla such as *Proteobacteria*, *Acidobacteria*, and *Bacteroidetes* play key roles in nutrient cycling, while complex root–microbe interactions shape the community composition and ecosystem functions [8–10]. Soil microbes further facilitate nutrient acquisition and promote tree growth, thus influencing height and DBH development.

In contrast to agricultural practices, the lack of soil management means that trees serve as a consistent food supply, fostering beneficial microbial populations as well as supporting various root diseases and parasites. Furthermore, soil bacteria can have detrimental impacts on plant development, thereby compromising the overall sustainability of forests and plantations. Pathogenic bacteria may induce diseases, disturb ecological equilibrium, and impede resistance. Compromised resilience can lead to the influx of harmful diseases that disrupt ecosystem balance [11]. In addition, under nutrient-deficient situations, soil bacteria may compete with plants for nutrients, thus inhibiting growth and productivity [12]. Severe soil erosion and degradation may lead to diminished microbial diversity and activity, compromised soil structure, and heightened vulnerability to abiotic stress [13]. Understanding the intricate relationship between soil bacteria and tree growth is pivotal in modern ecological research.

This work aimed to evaluate the biotic and abiotic properties that affect hardwood tree growth. In recent years, this relationship has emerged as a dynamic and intricate phenomenon, drawing increased attention from scientists and environmentalists alike. Our prior work showed that tree species influence microbial community composition [12]. Therefore, a deeper understanding of the microbes driving plant development would provide new insights to improve plant sustainability. We hypothesize that differences in soil prokaryotic community composition and interactions are associated with the growth performance of *Q. rubra* (red oak) and *J. nigra* (black walnut), such that healthier trees present a microbiota compared with poorly growing trees. To test this hypothesis, we analyzed the combined effects of soil properties and soil prokaryotic communities on the development of *Q. rubra* (red oak) and *J. nigra* (black walnut) across three sites in the central hardwood region (CHR) of Indiana and Michigan. In addition, we performed a co-occurrence network analysis to determine whether plant growth influences the relationship among soil prokaryotic taxa.

## Materials and methods

### Site description and experimental design

Our study was composed of a replicated multi-site field trial with species-specific plantations of *Q. rubra* and *J. nigra* planted in separate but comparable plantation plots at three locations using a uniform randomized complete block design with identical experimental structure across sites. Soils were collected from six *Juglans nigra* and *Quercus rubra* plantations located across three research sites in the central hardwood region of the United States: Western Michigan (42.7892° N, 85.5167° W), Southern Indiana (39.0341° N, 85.5128° W), and Western Indiana (40.4167° N, − 86.8752° W). Each plantation was established between 2002 and 2008 as a replicated field trial with uniform planting density (~ 3 × 3 m) on Rockfield, Crosby–Miami, Blount, and Ryker–Muscatatuck silt loam soils, respectively. Slope gradients ranged from 1% to 6%. The Michigan site included adjacent oak and walnut plantations separated by a narrow buffer zone. The plantations at the Indiana sites were spatially separated within their respective landscapes. No fertilizer or microbial amendments were applied to prevent interference with the native soil microbiome.

The Michigan site recorded an average annual precipitation of approximately 940 mm and an average annual temperature of 9.5 °C, based on data from U.S. Climate Data (https://www.usclimatedata.com). Annual snowfall was approximately 350 mm, and temperature extremes ranged from − 6 °C to 30 °C. The Western Indiana sites recorded an average annual temperature of 10.5 °C, with temperatures ranging from − 9 °C to 29 °C, approximately 1025 mm of annual precipitation, and about 500 mm of snowfall. The Southern Indiana sites recorded an average annual temperature of 12.5 °C, with temperatures ranging from − 2 °C to 27 °C, approximately 1041 mm of annual precipitation, and about 508 mm of snowfall. Precipitation at all sites followed a seasonal pattern with higher values in late spring (May–June). Climate data were obtained from on-site weather stations, Campbell Cloud (https://campbellcloud.io/map_index.php), and U.S. Climate Data (https://www.usclimatedata.com).

### Tree selection, growth assessments, and treatments

*J. nigra* and *Q. rubra* trees were established in species-specific plantations at each site under field conditions and were reliant on natural rainfall (Fig. S1) and existing soil characteristics (Figs. S2 and S3). Tree growth was assessed over multiple years, with final height and diameter at breast height (DBH) measured in November 2022. For both species and across all locations, individual trees were classified into two growth performance categories—high-performing (healthy) and low-performing (poor)—using the upper and lower 20% of DBH and height distributions within each block to define clearly divergent growth phenotypes while minimizing overlap between classes. Trees within the middle 60% of the distribution were not included in subsequent analyses.

The walnut plantations were classified as somewhat to moderately suitable for hardwood production using the walnut site suitability index [14]. A relative site suitability index was derived for oak by normalizing dominant height values across sites and scaling values between 0 and 1. Based on normalized index value distribution, the oak plantations were classified into ordinal suitability classes (low, marginal, moderate, and high suitability) with Western Michigan being highly suitable, Southern Indiana moderately suitable, and Western Indiana having low to marginal suitability. When possible, tree height measurements were collected at 5-, 10-, and 15-year intervals (H5, H10, H15), with site comparisons standardized to age 15. Dominant height (DH) was calculated as the mean height of the upper quartile (25%) of trees within each site at age 15. Diameter at breast height (DBH) was recorded as a supplementary growth variable but was not included in the suitability index. Site-level mean growth metrics were also calculated for comparison across plantations using the final DBH and height measurements collected in November 2022. Overall, the average height and DBH were additionally determined for each site and for healthy and poor growth blocks, and absolute growth rate (AGR) and relative growth rate (RGR) were calculated for healthy and poor blocks across available measurement intervals to support comparisons of growth performance among plantations (Fig. S4, Tables S1 and S2).

### Soil sampling

Soil sampling was conducted in November 2022. Six 20 × 20 m blocks separated by ≥ 50 m to capture within-site heterogeneity were established. Within each block, 25 soil cores (3 cm diameter, 0–10 cm depth) were collected and combined into composite samples following surface litter removal. This yielded eight composite replicates per site–species–growth class combination (*n* = 96 samples). Samples were ground manually to produce a powdered material, then sieved (2 mm) to improve sample homogeneity before being partitioned for molecular diagnostics and physicochemical analyses. Subsamples were stored at − 20 °C for DNA extraction or air-dried for chemical analyses.

### Soil physicochemical analysis

Soil physicochemical parameters were measured at the University of Missouri’s Soil Health Assessment Center (https://soilhealth.missouri.edu/). Soil texture was determined using laser diffraction analysis [15]. Soil pH, measured in water (1:5 soil: water) and 0.01 M calcium chloride (CaCl₂, soil: solution ratio = 1:2.5), and electrical conductivity (EC) were determined following protocols from Sparks and Page [16]. Soil organic carbon and nitrogen were quantified using elemental combustion analysis [17]. Soil moisture was determined using time- and frequency-domain reflectometry (TDR/FDR) [18]. Cation exchange capacity (CEC), extracted using NH4OAc (1 M, pH 7)/KCl (1 M), was measured using ammonium acetate extraction followed by ICP-OES analysis. Bulk density was determined using standard core sampling and computed tomography (CT) scanning [19].

### Microbial community profiling and bioinformatic analysis

Soil DNA was extracted using a commercial soil DNA extraction kit and used for bacterial 16 S rRNA gene amplicon sequencing and targeting the V4 hypervariable region using universal primers 515 F (5′–GTGCCAGCMGCCGCGGTAA–3′) and 806R (5′–GGACTACHVGGGTWTCTAAT–3′). PCR amplification was performed in triplicate using standard reaction conditions, followed by amplicon purification and Illumina library preparation. Sequencing was done on an Illumina MiSeq platform (2 × 250 bp) and depth varied among samples before rarefaction (range: 3025 − 5581 reads per sample). Samples were rarefied to 3025 reads to account for uneven sequencing depth prior to alpha diversity analyses. A standard quality control pipeline processed raw sequence data using primer trimming, read merging, and chimera removal, before taxonomic assignment. Water was included as a extraction and sequencing control (Fig. S5).

Raw sequences were processed using the DADA2 workflow (version 1.16) to perform quality filtering, primer trimming, read merging, chimera removal, and amplicon sequence variant (ASV) inference [20]. Default settings were used but modified to a minimum read length of 270 bp and maximum expected error threshold of 3 for both forward and reverse reads before they were combined to eliminate chimeric sequences using the consensus technique. Finally, the 16 S RNA sequences were aligned and taxonomic classification was performed against the SILVA v138.2 (https://www.arb-silva.de/) curated reference database [21].

### Statistical analysis

The statistical analysis was conducted in R version 4.2.0 (R Core Team, 2023). Growth categories were used for grouping, while soil and microbial variables remained continuous. Soil nutrient content and microbial community diversity (Shannon alpha-diversity index; alpha-diversity) were analyzed using ANOVA and Tukey’s post hoc test (*stats*, v4.3.0, and *agricolae*, v1.3-7, packages). The ASV count matrices, taxonomic assignment, and sample metadata were combined to create ‘phyloseq’ objects with the “phyloseq” package (v1.46.0) [22], and the graphics were generated using the ‘ggplot2’ package [23]. Shannon alpha-diversity index was calculated using the function *estimate_richness* (*phyloseq* package, v1.46.0) based on the rarefied abundance.

Nonmetric dimensional scaling (NMDS) ordination based on relative abundance was used to evaluate the microbial structure. For this analysis, percentage-transformed ASV count data for DNA were used, and the Bray-Curtis dissimilarity index was computed to generate a distance matrix between the samples using the “vegan” package [24]. The NMDS multivariate analyses were plotted using the ‘ggplot2’ package. Effects of explanatory variables on microbial community composition were assessed by permutational multivariate analysis of variance (PERMANOVA) using the function adonis2 in the ‘vegan’ package. PERMANOVA models included site, tree species, plant state, and all interactions as explanatory variables. Pairwise PERMANOVA was done using the function *pairwise.adonis* in the ‘pairwiseAdonis’ package [25]. Microbial communities under tree species and plant state were analyzed at a low taxonomic resolution (phylum and genus level) to characterize overall community shifts. The differential abundance of relative abundance data for bacterial phyla and genera was examined using *DESeq2*, with plant state as a fixed effect categorized by location and tree species. Taxa present in fewer than 1% of samples were excluded before analysis. Differentially abundant taxa were identified based on Bonferroni-Holm-adjusted p-values (i.e., q-values (q)) [26].

Mantel test and linear correlation were used to associate soil parameters and bacteria with the plant’s growth. Mantel tests were used to evaluate concordance among distance matrices derived from microbial community composition, soil properties, and plant growth metrics. Multiple comparisons and Bonferroni adjustments were then performed to determine any variations in community makeup (adonis function, vegan package). The NMDS1 axis was used as a summary variable representing dominant variation in microbial community composition.

The SpiecEasi package (v1.0.0) in R was used to examine microbial co-occurrence patterns across tree species, site, and plant growth development. Correlations between genera were estimated with the sparCC function within SpiecEasi [27, 28]. Genera with a frequency below 75% and sparCC correlation values of 0.6 or below were excluded prior to network construction. Network complexity was assessed based on average connectivity, clustering coefficient, and edge number calculated with the vegan and igraph packages (v1.6.0) in R [29] and followed established microbial co-occurrence approaches [30]. Each network was constructed using 8 replicates; then the network parameters were analyzed using the site as replicates and using ANOVA and Tukey’s post hoc test (*stats*, v4.3.0, and *agricolae*, v1.3-7, packages).

Finally, Random Forest classification models were implemented using the randomForest package (v4.7-1.1) to identify those taxa and soil properties that most strongly influence the tree species growth [31]. The out-of-bag (OOB) error was calculating using the randomForest package [31]. The Random Forest mean significance was calculated using the rfPermute package (v2.5.4) [32]. Furthermore, the R scripts used for data analysis and processing are publicly available at https://github.com/JuanFrene/CHR-Bacteria.

## Results

### Hardwood tree growth and development

**Fig. 1:**
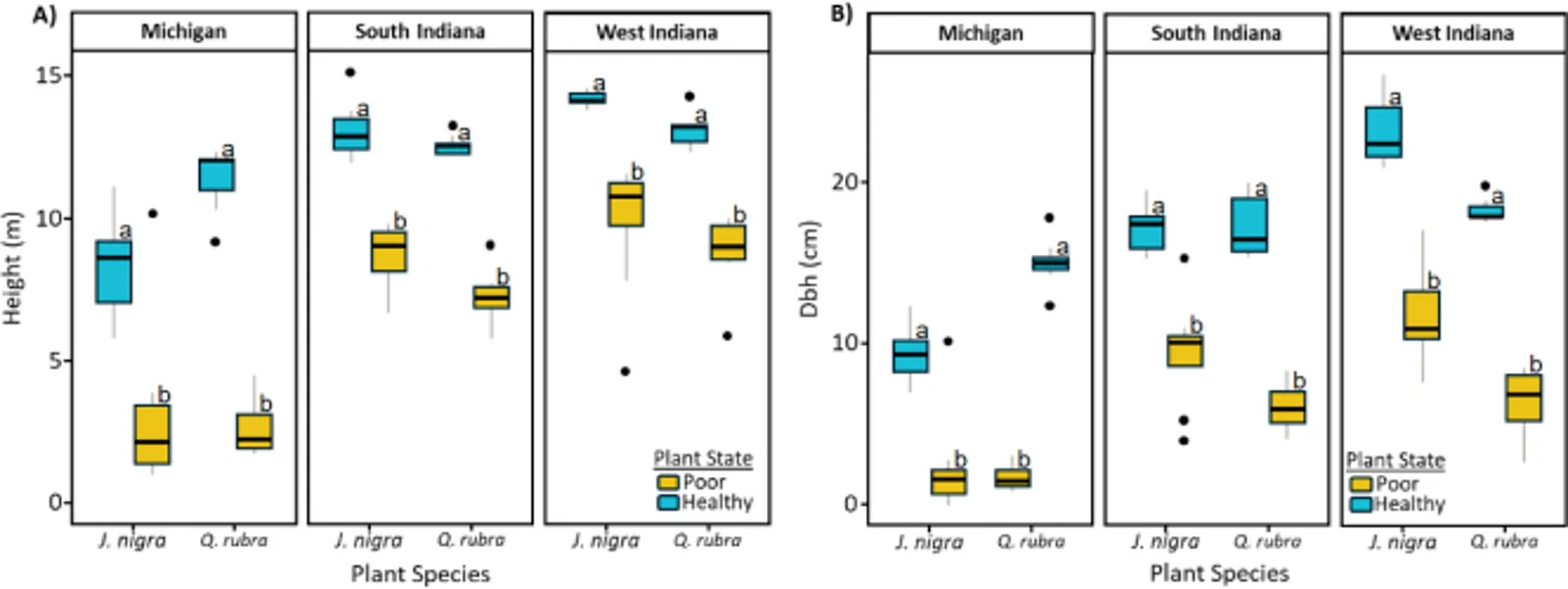
Hardwood tree growth. Boxplot of **A** tree height and **B** diameter at breast height (DBH) for the three sites, both species (*J. nigra* and *Q. rubra*), and the plant development state (healthy and poor). Mean values + standard deviation. Data were tested for significance by one-way ANOVA followed by Tukey’s post hoc test (*P* < 0.05). (*n* = 8)

The measurements used to assess tree growth were mean dominant height and stem diameter (DBH), and both measurements were significantly correlated (R^2^ = 0.8046, *P* < 0.001). High- and low-performing trees differed in mean height and DBH across sites. Larger sizes were observed in Southern and Western Indiana, and smaller sizes in Michigan (Fig. 1a and b). Although *J. nigra* tended to be larger than *Q. rubra* in Indiana, the reverse was observed in Michigan, though these differences were not statistically significant (*P* > 0.05). The proportion of low-performing trees was 23% and 50% for *J. nigra* and 12% and 35% for *Q. rubra* in Michigan and Indiana, respectively (Fig. 1a and b). Differences between plant growth were generally more pronounced for DBH than for height measurements, particularly in *J. nigra* plots (Fig. 1a and b). Across most plots and measurement types, healthy high-performance trees exhibited greater AGR and RGR values than poor low-performance trees, indicating enhanced growth under favorable site conditions (Table S1).

### Soil properties on *J. nigra* and *Q. rubra* growth

**Fig. 2:**
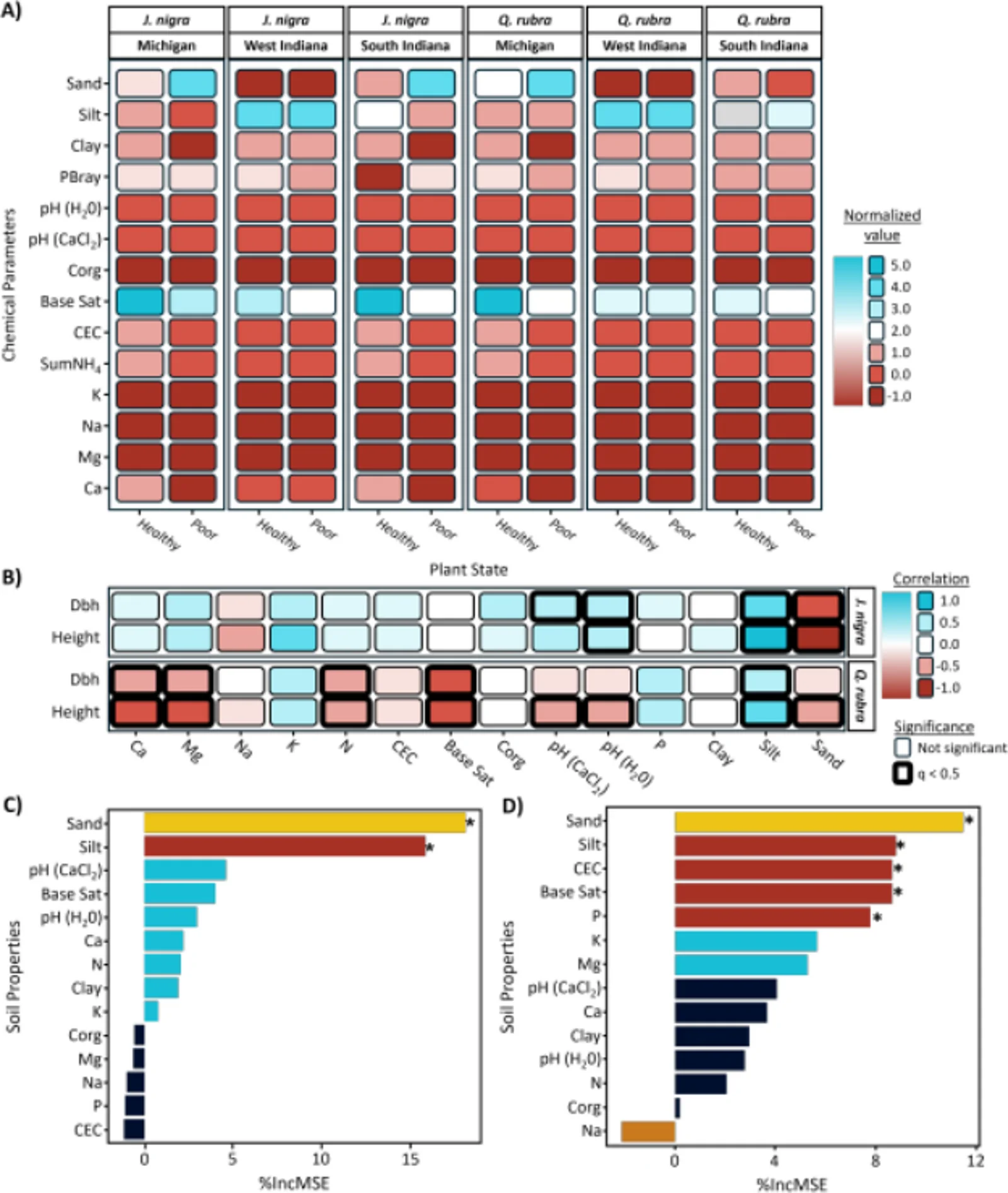
Soil chemical and physical parameters of the plantations. Heatmap showing **A** the normalized values of soil parameters for the three sites, both plant species (*Q. rubra* and *J. nigra*), and the plant development state (good and bad); **B** Pearson correlation between soil parameters and tree height and diameter at breast height (DBH). Random forest mean predictor importance (% of increase in mean square error) of soil properties and plant height for **C** *J. nigra* and **D** *Q. rubra*. This accuracy importance measure was computed for each tree and averaged over the forest (5000 trees). *Variables identified as the most significant predictors by the Random Forest model. (*n* = 8)

Soil parameters showed that organic carbon (Corg), organic N (Norg), and cation exchange capacity (CEC) varied significantly by species, but not by site or growth condition (Fig. 2a). Phosphorous did not differ across any grouping (Fig. 2a). Soil pH, measured by both H_2_O and CaCl_2_, was significantly lower in *Q. rubra* and poorly performing trees (Fig. 2a). Base saturation also differed significantly by species, location, and growth condition (Fig. 2a). We additionally measured several soil cations. Of those measured, magnesium (Mg) varied with the same factors as base saturation and was thus different by plant species, location, and growth condition (Fig. 2a). Calcium (Ca) concentrations were higher in *J. nigra* and potassium (K) was more abundant in Indiana than in Michigan (Fig. 2a). Soil texture analysis indicated substantial variations between Michigan and Indiana soil with Michigan soil being predominantly sandy whereas Indiana soils were silty (Fig. 2a).

When we analyzed the soil properties that influence plant development, *Q. rubra* showed stronger associations with more soil parameters than *J. nigra*. Specifically, *Q. rubra* development was negatively associated with Ca, Mg, Norg, soil pH, and sand content and positively associated with silt (Fig. 2b). In contrast, *J. nigra* growth was positively correlated with soil pH and silt and negatively associated with sand (Fig. 2b). However, no single soil parameter was clearly associated with developmental disparities within each site. Linear correlations indicated that soil pH, sand, and silt could account for site-specific differences in growth but not differences within sites (Fig. S6). We applied a Random Forest (RF) as an exploratory analysis to identify which soil properties most influenced height (Fig. 2c and d) and DBH (Fig. S7). For *J. nigra*, sand and silt were the most important predictors of height (Fig. 2c). For *Q. rubra*, sand, silt, CEC, base saturation, and phosphorus were the primary contributors (Fig. 2d).

### The effect of soil microbiota on plant species, location, and plant development

**Fig. 3:**
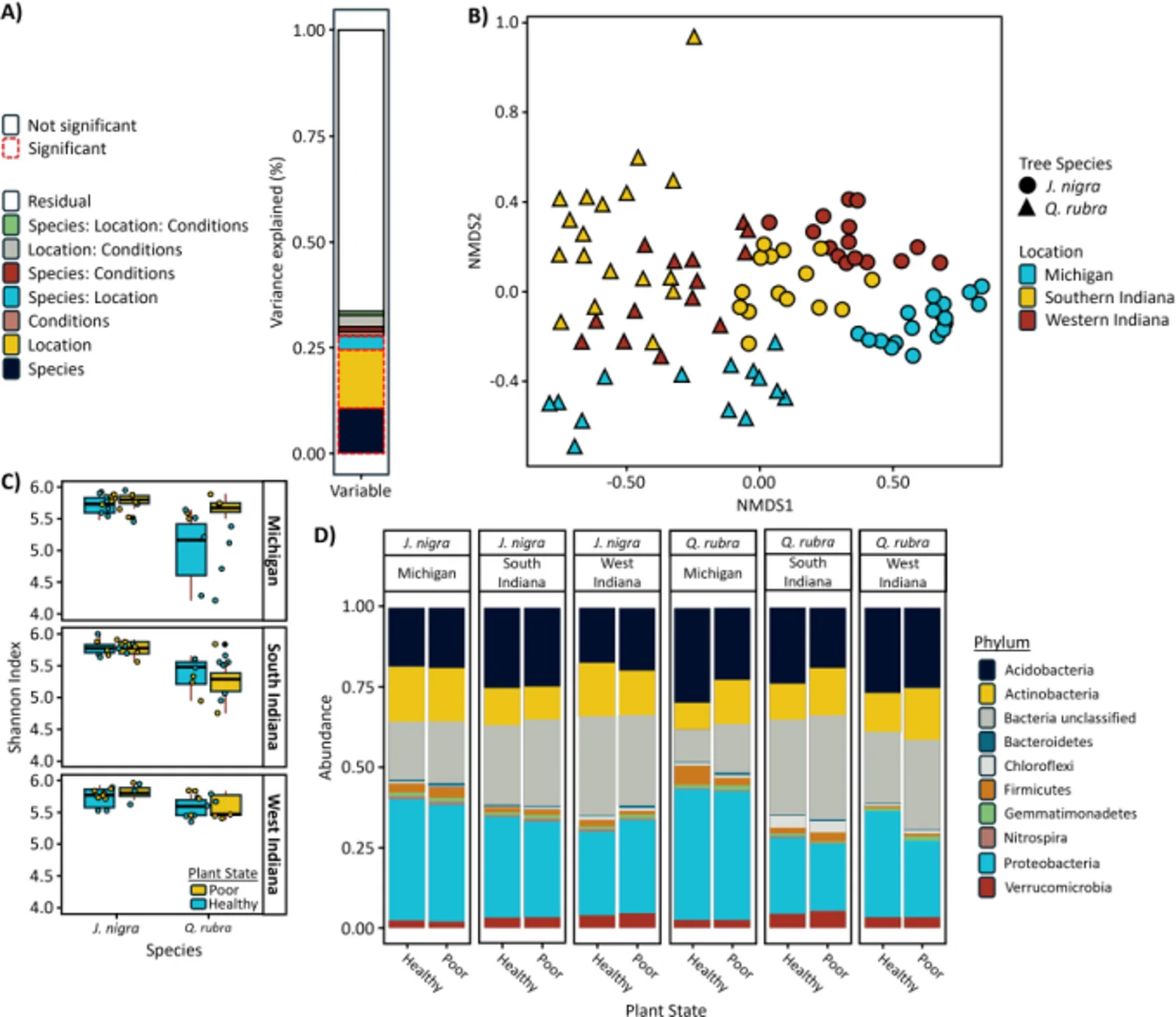
Soil bacterial structure of hardwood trees. **A** Permutational multivariate analysis of variance (PERMANOVA) analysis of the effect of site, plant species, and plant development state on the soil bacterial community. **B** Non-metric MultiDimensional Scaling (NMDS) based on the Bray-Curtis dissimilarity distance at the bacterial ASV level for the three sites, both plant species (*J. nigra* and *Q. rubra*), and the plant development state (healthy and poor). **C** The alpha-diversity index, measured as the Shannon index, shows the mean values + standard deviation. **D** Stacked bar plots indicate the relative abundance (%) of the 10 main bacterial phyla. (*n* = 8)

We characterized the bacterial/archaeal communities via 16 S rRNA sequencing across all plant species and sites, rarefying to 3025 ASVs per sample. The PERMANOVA analysis showed that the prokaryotic communities differed significantly by tree species (10.7% of variance), location (9.4%), and their interaction (7.57%) (Fig. 3a). In contrast, community composition did not differ significantly among tree growth conditions (Fig. 3a). The pairwise PERMANOVA analysis by site showed significant differences between the three sites (Table S3). The Non-metric MultiDimensional Scaling (NMDS) analysis indicated that the samples were differentiated by tree species identity along the NMDS1 axis, whereas location was correlated with the NMDS2 axis (Fig. 3b). *Q. rubra* samples were situated along the negative axis of NMDS1, while the samples from Michigan were positioned along the negative axis of NMDS2 (Fig. 3b). Alpha diversity was significantly higher in *J. nigra* than in *Q. rubra*, and it was higher in western Indiana than in southern Indiana (Fig. 3c). An interaction between plant development and site was observed. For example, in Michigan, low-performing trees showed higher alpha diversity, whereas in Indiana, *J. nigra* showed consistently higher alpha diversity values regardless of growth class (Fig. 3c). No significant correlations were observed between height or DBH and Shannon index (or any other alpha diversity indices) (Fig. S8). Communities were dominated at the phylum level by *Proteobacteria*, *Acidobacteria*, and *Actinobacteria* (Fig. 3c). In contrast, the genus level was mainly dominated by *Gp1*, *Gp3*, *Gp6*, and *Gp16* (*Acidobacteria*), *Spartobacteria incertae sedis* (*Verrucomicrobia* phylum), and *Bradyrhizobium* (*Proteobacteria* phylum) (Fig. 3d).

To analyze how plant growth development affected the structure of soil prokaryotic communities, co-occurrence networks were constructed using species, plant growth, and site as replicates (Table 1). The number of nodes and edges was determined by plant species, being significantly higher in *J. nigra* (Table 1). In addition, the average degree was also significantly higher in *J. nigra*. When we compared modularity, mean distance, edge density, and transitivity, there was an opposite trend across tree species: *Q. rubra* showed lower values in high-performing trees, while J. nigra showed the opposite trend (Table 1).

**Table 1:** Topological features of the co-occurrence networks constructed based on the prokaryotic community based on tree species and plant growth development

Tree Species	Plant Growth	edges	nodes	CC	spl	md	gd	nd	ad
*Q. rubra*	Healthy	769.33b	62.66b	0.407	1.595	0.086	0.402	2.00	24.48b
	Poor	903.33b	64b	0.443	1.557	0.079	0.442	2.00	28.09b
*J. nigra*	Healthy	1273a	78.66a	0.420	1.584	0.082	0.415	2.00	31.74a
	Poor	1224.33a	81.66a	0.375	1.623	0.087	0.373	2.00	29.9a

Values with different letters are significantly different among seasons (*p*-value < 0.05; ANOVA, followed by Tukey post-hoc test). (*n* = 3)

**Fig. 4:**
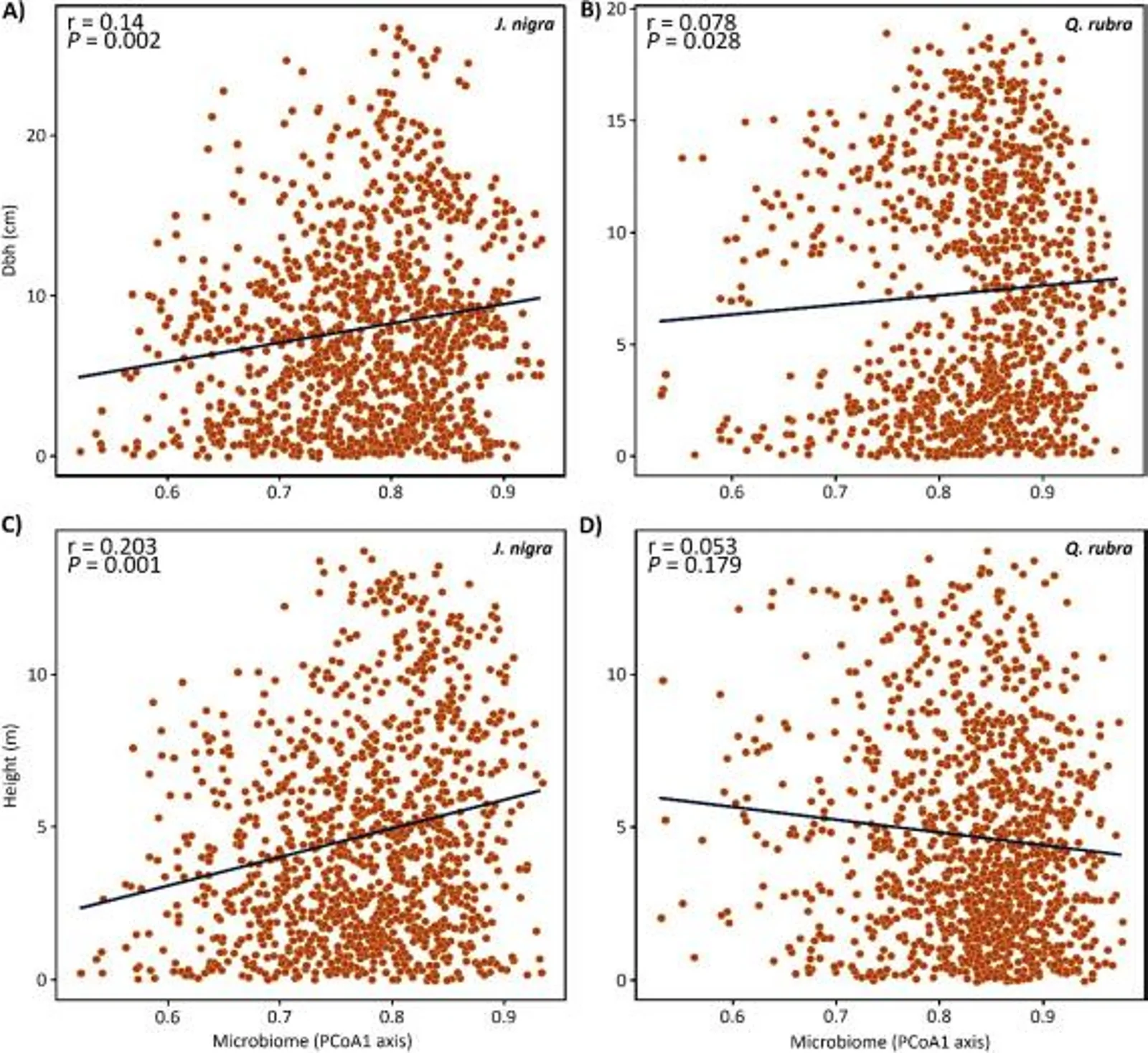
Association between microbial structure and plant growth. Mantel test correlation between soil bacterial structure, measured as axis 1 of the Bray-Curtis dissimilarity distance at the bacterial ASV level, DBH (**A**, **B**), and height (**C**, **D**). Plant growth parameters for *J. nigra* (A, C) and *Q. rubra* (**B**, **D**)

When we analyzed the relationship between the prokaryotic community and plant growth, height, and DBH. Tree DBH was significantly correlated with microbiota composition in both species (*J. nigra*: Mantel *r* = *0.14*, *P* = *0.002*, Fig. 4a; *Q. rubra*: Mantel *r* = *0.078*, *P* = *0.028*, Fig. 4b). Height was only correlated with *J. nigra* and not *Q. rubra* (Mantel *r* = *0.20*, *P* = *0.001*, Fig. 4c and d). Additionally, soil properties also significantly correlated with microbiome structure for both plant species (*J. nigra*: Mantel *r* = *0.55*, *P* = *0.001*; *Q. rubra*: Mantel *r* = *0.37*, *P* = *0.001*, Fig. S9). The microbial community associated with *Q. rubra* correlated with Mg, Ca, K, N, CEC, base saturation, pH (H_2_O and CaCl_2_), clay, silt, and sand (Table 2). The prokaryotic community associated with *J. nigra* only correlated with base saturation, clay, silt, sand, P, and K (Table 2).

**Table 2:** Table of correlation between microbiome structure based on PCoA1 of *J. nigra* and *Q. rubra* and soil parameters

Properties	J. nigra		Q. rubra	
	*R*	*p*-value	*r*	*p*-value
Mg	0.0623	0.091	0.2652	0.001
Ca	0.0398	0.192	0.3142	0.001
K	0.2698	0.001	0.1188	0.002
N	0.0394	0.19	0.3169	0.001
CEC	0.0486	0.147	0.282	0.001
Base_Sat	0.241	0.002	0.274	0.001
Corg	0.038	0.283	0.0191	0.289
pH CaCl2	0.058	0.16	0.1975	0.001
pH H2O	0.0648	0.084	0.1759	0.001
P	0.07685	0.061	0.06074	0.153
Clay	0.122	0.01	0.1993	0.003
Silt	0.5803	0.001	0.3376	0.001
Sand	0.5267	0.001	0.3632	0.001

### Which members of the prokaryotic communities can be linked with plant growth and development?

**Fig. 5:**
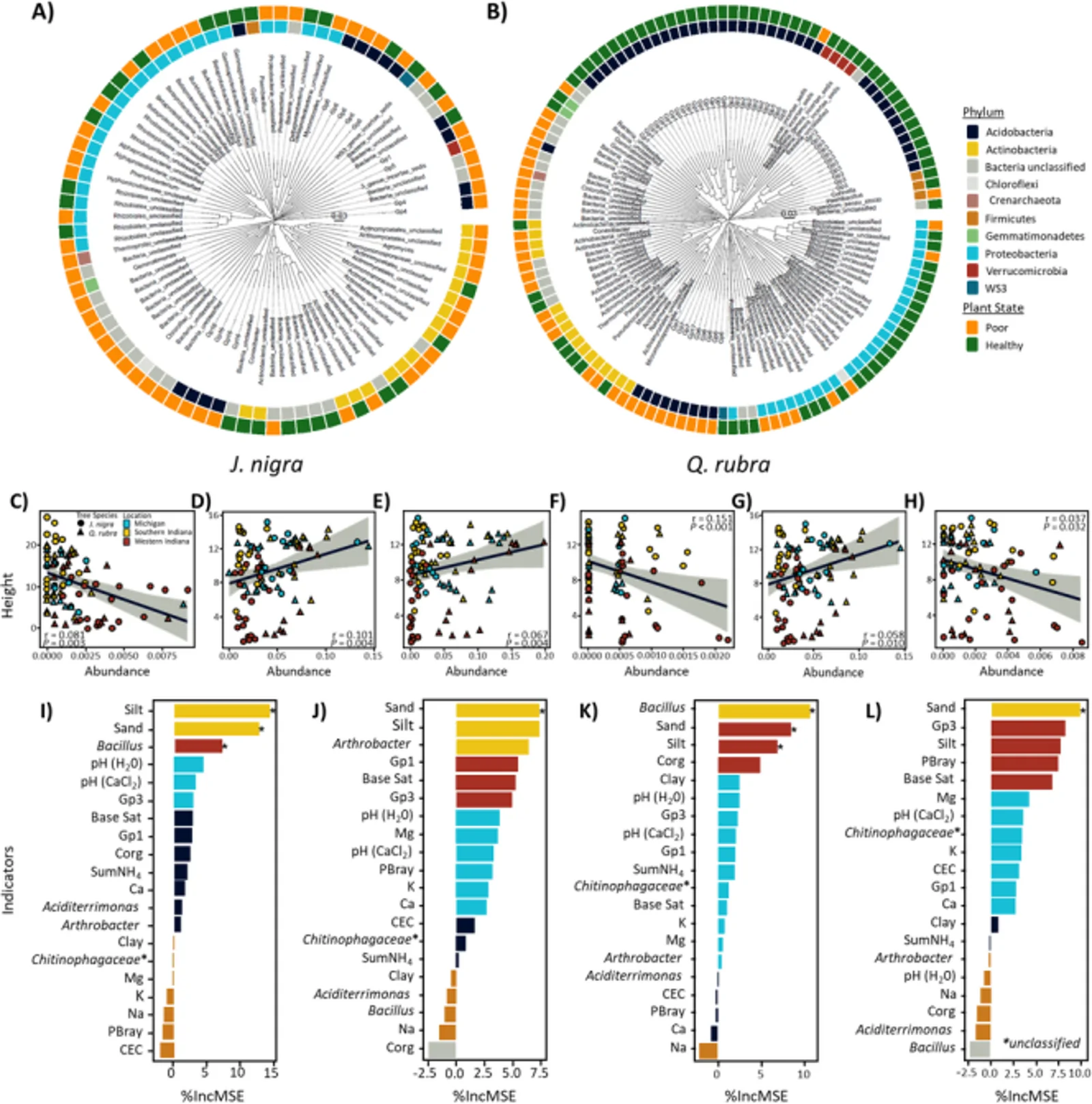
Bacterial relationship between plant growth and soil bacterial genus. Tree showing the ASV with differential abundance between plants with high- and low-performing trees for **A ***J. nigra* and **B ***Q. rubra.* Dot plot showing the Spearman correlation between plant height and **C ***Bacillus*, **D ***Gp3*, **E ***Gp1*, **F ***Aciditerrimonas*, **G ***Arthrobacter*, **H ***Chitinophagaceae_unclassified*. Random forest mean predictor importance (% of increase in mean square error) of soil properties and plant height and DBH for *J. nigra* (Fig. 5i and k) and *Q. rubra* (Fig. 5j and l). This accuracy importance measure was computed for each tree and averaged over the forest (5000 trees). (*n* = 8)

Comparisons of ASVs between high-performing (healthy) and low-performing (poor) trees revealed 88 (67%) differentially abundant ASVs in *J. nigra* and 122 (47%) in *Q. rubra* (Fig. S10). Additionally, we found 59 (*J. nigra*) and 45 (*Q. rubra*) ASVs that were more abundant in low-developing individuals (Fig. 5a and b). The most responsive phyla were *Acidobacteriodota* in *Q. rubra* and *Proteobacteria* in *J. nigra* (Fig. 5a and b). Within *J. nigra*, *Betaproteobacteria* were twice as prevalent in low-performing trees (30%) compared to those with high-performing trees (18%) (Fig. 5a). Within *Q. rubra*,* GP1*, *GP2*, and *GP3* were most abundant in high-developing trees, while *GP6* and *GP7* were predominant in low-developing individuals (Fig. 5b).

We then examined which taxa correlated with height and DBH and identified 56 and 45 taxa, respectively. Within these taxa, those with > 50% prevalence and significant correlations were: *Aciditerrimonas* (Height: *r* = 0.081, *P* = 0.003; DBH: *r* = 0.047, *P* = 0.018) (Fig. 5c, Fig S10d), *Arthrobacter* (Height: *r* = 0.101, *P* = 0.004; DBH: *r* = 0.064, *P* = 0.007) (Fig. 5d, Fig S10e), *Chitinophagaceae unclassified* (Height: *r* = 0.067, *P* = 0.004; DBH: *r* = 0.047, *P* = 0.019) (Fig. 5e, Fig S10f), *Bacillus* (Height: *r* = 0.151, *P* < 0.001; DBH: *r* = 0.145, *P* < 0.001) (Fig. 5f, Fig S10a), *Gp3* (Height: *r* = 0.058, *P* = 0.010; DBH: *r* = 0.038, *P* = 0.048) (Fig. 5g, Fig S10b) and *Gp1* (Height: *r* = 0.037, *P* = 0.032; DBH: *r* = 0.030, *P* = 0.048) (Fig. 5h, Fig S10c).

Finally, we re-ran the Random Forest (RF) analysis, including both soil variables and the newly identified microbial taxa (Fig. 5i, j, k and l). The OOB error was calculated, and it showed a decrease in magnitude for height, but not for Dbh (Table S4). The updated RF confirmed that sand and silt remained the strongest predictors of plant growth. Additionally, microbial taxa such as *Bacillus* and *GP3* are two taxa that explain part of the variation in the height of *J. nigra*, in conjunction with soil pH (Fig. 5i). *Arthrobacter*, *GP1*, and *GP3* are the main bacterial taxa that explain part of the variation in *Q. rubra* height (Fig. 5j). However, *Bacillus* appeared as the best parameter to explain DBH in *J. nigra* (Fig. 5k) and *GP3* as the second for DBH in *Q. rubra* (Fig. 5l).

## Discussion

This study showed that site-specific conditions, mainly determined by site location, and specific microbiota members, as we hypothesized, showed a greater association and correlation with plant growth and development. The influence of soil chemical properties, such as Na or K, or the overall prokaryotic community structure, had less weight in our exploratory models. In this work, key taxa associated with plant development vary with hardwood tree species and are independent of the parameters used to measure tree growth (height or DBH). While previous forest studies have emphasized the dominant role of soil chemistry in shaping plant productivity, our results suggest that key microbial taxa and soil physical structure may exert more direct control over hardwood growth under relatively homogeneous climatic conditions [33]. The observed differences in soil and microbial characteristics reflect contrasts in growth performance under comparable site conditions rather than differences among sites because high- and low-performing blocks were selected within each site based on relative height and DBH distributions. All walnut plantations were classified as somewhat to moderately suitable for hardwood production according to the Wallace and Young Soil Index [14], indicating broadly comparable site quality among locations. This suggests that observed growth differences were unlikely to be driven solely by large differences in inherent site productivity. Within this context, oak plantations exhibited a clear productivity gradient based on dominant height structure, with Western Michigan reflecting comparatively higher suitability conditions, Southern Indiana intermediate conditions, and Western Indiana lower to marginal conditions.+ In addition, analyses treating height and DBH as continuous variables yielded consistent relationships with soil and microbial parameters, indicating that the observed patterns were not an artifact of dichotomizing growth classes. Additionally, hardwood trees with low growth development showed a differential soil bacterial community compared to high-growth development plants for both species, *J. nigra* and *Q. rubra*.

In this study, the primary soil factors influencing plant development were soil properties such as sand, silt, and pH. *J. nigra* is a highly demanding species in comparison with *Q. rubra* and needs a moderate or moderately light texture like sand-loam, loam-sand, or, preferably, loam, and a high requirement for Ca, Mg, K, and a moderate need for N [34]. On the contrary, *Q. rubra* is a less demanding species, suitable for poor (low demand for N, P, and K), acidic, and heavy or light soils [35]. Our findings clearly illustrate these species-specific requirements, as *J. nigra* exhibited positive trends with soil nutrients, whereas *Q. rubra* showed negative and significant correlations with Ca, Mg, and N. The significant correlation between the growth and development of *J. nigra* and sand content lies in its sensitivity to waterlogging and soil compaction; hence, this species requires soil with low to moderate texture and a high sand content for optimal growth. The association between plant growth and soil characteristics has been previously studied [36–38]. Finally, Toledo et al. [39] showed that soil properties have less influence on tree growth rate compared to climate (rainfall and temperature) in Bolivian forests. Here, hardwood trees were classified into contrasting growth groups (high and low) based on block-level DBH and height distributions within each site. Although plantation establishment years differed among sites, growth categories were assigned relative to local site conditions rather than absolute growth values. Therefore, observed differences in development are more likely associated with local soil and microbial variation than with broad climatic differences among individuals.

Plant roots shape the soil into a biologically active zone characterized by intense interactions among soil microbes, nutrients, and root-derived compounds. The relationship between plants, soil, and microorganisms is reciprocal and may influence both plant and microbial community dynamics [40]. Plants can alter the microbial communities that develop around their roots through the release of exudates and other metabolites. Some of the taxa observed in these environments have been linked in previous studies to nutrient cycling, plant hormone production, and suppression of plant pathogens [41]. In the present study, microbial activity was not directly measured. Therefore, these processes should be considered possible explanations rather than confirmed mechanisms. Our results showed that both species, *J. nigra* and *Q. rubra*, have differential root microbiomes that are independent of location. These differences may reflect plant–soil–microbe feedback processes. Similar patterns have been reported previously [42, 43]. For example, the *Q. rubra*-associated bacterial community showed greater abundance of *Acidobacteria*, *Chloroflexi*, and *Firmicutes* [44]. *Acidobacteria* are often reported to be associated with acidic soil conditions in forest ecosystems. The greater abundance observed in *Q. rubra* soils could reflect differences in local soil conditions, such as pH, as has been the case in other studies [45, 46]. This study did not test the mechanism responsible for that pattern, so this interpretation remains tentative. On the contrary, differences in microbial communities associated with *J. nigra* might be linked to variation in root-derived carbon sources or secondary metabolites previously reported for this species, possibly because of rich C exudates like juglone [47]. Exudate composition was not measured in this study. As a result, this explanation should be viewed as a working hypothesis rather than direct evidence. Future investigations on the exudate profile of *J. nigra* would help clarify potential interactions between juglone and soil microbial populations.

Soil microorganisms coexist within complex ecological networks. Co-occurrence network analysis revealed clear differences between tree species, with *J. nigra* samples exhibiting larger networks and a greater number of connections than *Q. rubra* samples. Increased network connectivity in *J. nigra*-associated communities may reflect enhanced metabolic interdependence among taxa, potentially facilitating nutrient turnover in nutrient-rich microsites. However, greater connectivity may also result from shared environmental filtering rather than direct biological interactions, which is a recognized limitation of correlation-based network analyses. The greater connectivity observed in *J. nigra* soils may be related to their enriched C and N environment, which likely provides a broader range of energy sources for soil microbiota and may contribute to the higher alpha diversity observed in these samples (Fig. 3c). However, the relationship between plant growth condition and network structure differed between tree species. Healthy *Q. rubra* trees exhibited higher average path length and mean degree values, indicating greater connectivity among microbial taxa in soils associated with better tree performance. Previous studies have similarly reported that greater plant development is associated with larger and more complex microbial co-occurrence networks [48]. Conversely, the increase in network complexity observed in poorly developed *Q. rubra* individuals may be associated with distinct soil properties, particularly soil pH and Na content, which were identified as important factors related to plant development (Fig. 2). Nevertheless, the consistent differences observed between tree species suggest that host identity plays an important role in structuring microbial interaction networks.

Soil microbial structure and abundance are influenced not only by plants through exudates and litter but also by soil properties like pH, phosphorus, and nitrogen concentrations. Notably, overall alpha- and beta-diversity metrics were not associated with plant growth, whereas specific taxa exhibited consistent correlations. This finding challenges the common assumption that greater microbial diversity necessarily promotes plant productivity. Instead, our results support the hypothesis that particular functional groups, rather than whole-community diversity, drive host performance in hardwood systems. In this way, there is three-way feedback between soil, plants, and microbes. The prokaryotic community showed a significant correlation with soil chemical properties for both tree species during our study (Fig. 3). Following this analysis, we analyzed each soil chemical and physical parameter with each tree species-associated microbial community, revealing that *Q. rubra* exerted a more significant effect on soil parameters affecting soil bacteria than the *J. nigra*-associated microbiome. Though the present study did not evaluate this directly, *Q. rubra* litter has been reported to contain higher lignin and phenolic concentrations and altered C/N ratios, which may influence carbon and nitrogen cycling and contribute to differences in microbial community structure [49]. Previous studies suggest that *Q. rubra* may modify some soil conditions that are the main drivers of soil microbial communities, like pH [43, 45]. On the contrary, previous reports from *J. nigra* studies suggest that root-derived inputs differ in chemical composition and microbial accessibility [47]. Those differences may contribute to variation in soil microbial communities; however, this interpretation is speculative as we did not investigate exudate production and uptake.

In this study, hardwood trees were classified according to different growth and development based on DBH and plant height into two groups, identified as healthy and poor based on block-level height and DBH distributions within each site. Growth–environment relationships were also evaluated using continuous height and DBH measurements. The observed microbial and soil associations are more likely to reflect within-site variation in tree development than broad environmental differences among locations because growth classes were defined relative to local site distributions rather than absolute performance thresholds. According to our results, the prokaryotic community within these two groups exhibited no significant differences, and we could not correlate the entire bacterial community (alpha or beta diversity) with plant development. However, we found that specific taxa correlated with plant growth [49], and those taxa differed for each tree species. Previous studies have suggested that individual bacterial taxa may be more strongly associated with plant growth or root development rather than the entire microbial community [50, 51]. Members of the *Bacillus* genus have been associated with plant growth promoters and synthesis of antibiotics against pathogens, though it is pertinent to emphasize that *Bacillus* comprises a vast group and not all members exert beneficial effects [52]. Sun et al. [53] reported that some members of *Bacillus* can also stimulate other plant-beneficial microbes like *Pseudomonas protegens*. Similarly, taxa within *Arthrobacter* have been associated with plant growth, nutrient uptake, and stimulation of stress responses in previous studies [54, 55]. Other important taxa identified included the *Acidobacteria* subgroups GP1 and GP3, previously reported in association with ecological conditions linked to pinewood nematode suppression [56]. However, these functional roles were not evaluated in the present study.

A significant drawback of this work is the sample size supporting various analyses, with most comparisons performed using *n* = 8 per group across tree species, locations, and plant states. This degree of replication is typical in field-based microbial ecology research; however, it inherently constrains statistical power, heightens uncertainty in effect size estimations, and may augment the likelihood of false positives, especially in multivariate, distance-based, and machine-learning methodologies. Consequently, results from PERMANOVA, Mantel tests, differential abundance analysis, and Random Forest models must be read judiciously. Random Forest studies primarily serve as exploratory instruments to identify potentially important characteristics rather than to provide conclusive predictive models, and their efficacy may be susceptible to sampling variability under the current design. We thereby underscore the need for consistent patterns across several analytical methodologies and soil depths, rather than depending on a single test. Further research will be crucial to corroborate and expand the results of this study.

## Conclusion

Overall, this study found that site-specific soil type and individual microbiota members were strongly associated with patterns of plant growth and development. The key bacterial taxa linked to plant growth drivers included *Bacillus*, unclassified *Chitinophagaceae*, *Aciditerrimonas*,* Arthrobacter*, *GP1*, and *GP3.* The taxa identified in relation to this study are widely known for their involvement in nutrient cycling and the plant immune system response against pathogens, though identification of specific functional roles was not investigated in this study. A systematic analysis of the microbiomes involved in the development of *J. nigra* and *Q. rubra* may help improve understanding of plant–soil–microbe relationships in hardwood systems. Such an approach may also provide a new strategic framework for future hypothesis-driven studies of microbial contributions to tree growth. These findings highlight the complexity and species-specific nature of plant–soil–microbe interactions in hardwood ecosystems and suggest that within-site variation in growth performance is associated with distinct microbial community signatures, independent of broad geographic location. Because this study is correlational in design, no direct causal inferences can be made regarding the role of individual microbial taxa in driving plant growth outcomes, and any implications for forest management or applied use of microbial indicators should be considered preliminary. More analysis is needed to validate the incorporation of microbial indicators into forest management strategies, which could enhance the predictive accuracy of growth performance under varied environmental conditions. Further experimental research is needed to validate the functional roles of these bacterial taxa and assess the feasibility of developing microbiome-informed approaches as practical tools in silviculture or forest management.

## Supplementary Information

Below is the link to the electronic supplementary material.

Supplementary Material 1

## Data Availability

The Sequence Read Archive (SRA) of the National Center for Biotechnology Information (NCBI) has all raw sequencing data stored under the BioProject number PRJNA1312248. Furthermore, the R scripts used for data analysis and processing are publicly available at [https://github.com/JuanFrene/CHR-Bacteria](https:/github.com/JuanFrene/CHR-Bacteria) .
