# Supplementary material for: Evaluating the role of biotic and abiotic factors in hardwood tree development in the Central Hardwood Region (CHR)
Source: Environ Microbiome. 2026 Jul 14;21:120. doi: 10.1186/s40793-026-00935-2 (PMC13651037; doi:10.1186/s40793-026-00935-2)
Supplement: Supplementary file 1 [file 40793_2026_935_MOESM1_ESM.docx]

Supplementary information for:

**Evaluating the role of biotic and abiotic factors in hardwood tree development in the Central Hardwood Region (CHR).**

Shaneka S. Lawson^1+^*, Juan P. Frene^2+a^, Niall Lue Sue^1^

^1^USDA Forest Service, Northern Research Station, Hardwood Tree Improvement and Regeneration Center (HTIRC), PFEN226C, Department of Forestry and Natural Resources, Purdue University, 715 West State Street, West Lafayette, IN 47907, USA

^2^ University of Nottingham, School of Biosciences, Sutton Bonington, Nottingham LE12 5RD, UK

^+^ Both authors equally contributed

^a^Currently at: Agricultural Science Center, New Mexico State University, Clovis, NM, 88101, USA

* Authors for correspondence: Shaneka S. Lawson, [shaneka.s.lawson@usda.gov](mailto:shaneka.s.lawson@usda.gov)

**Table S1.** Mean (± SEM) absolute growth rate (AGR) and relative growth rate (RGR) for high- and low-performance blocks of northern red oak and black walnut based on height and diameter at breast height (DBH) measurements. Growth intervals differed among plots according to measurement schedules and available assessment periods. AGR and RGR values are reported separately for height (m yr⁻¹) and DBH (cm yr⁻¹) growth metrics.

| **Species** | **Measurement** | **Plot Location** | **Interval (yr)** | **High AGR ± SEM** | **Low AGR ± SEM** | **High RGR ± SEM** | **Low RGR ± SEM** |
| --- | --- | --- | --- | --- | --- | --- | --- |
| Oak | Height | Western Indiana | 5–15 | 0.868 ± 0.020 | 0.622 ± 0.062 | 0.125 ± 0.014 | 0.125 ± 0.014 |
| Oak | DBH | Western Indiana | 5–15 | 1.382 ± 0.028 | 0.586 ± 0.065 | 0.138 ± 0.004 | 0.147 ± 0.017 |
| Oak | Height | Southern Indiana | 5–15 | 0.878 ± 0.026 | 0.478 ± 0.019 | 0.115 ± 0.004 | 0.115 ± 0.004 |
| Oak | DBH | Southern Indiana | 5–15 | 1.381 ± 0.064 | 0.511 ± 0.036 | 0.166 ± 0.008 | 0.361 ± 0.025 |
| Oak | Height | Michigan | 3–15 | 0.813 ± 0.031 | 0.147 ± 0.026 | 0.067 ± 0.012 | 0.067 ± 0.012 |
| Oak | DBH | Michigan | 3–15 | 1.163 ± 0.052 | 0.064 ± 0.011 | 0.251 ± 0.013 | 0.102 ± 0.025 |
| Walnut | Height | Western Indiana | 3–20 | 0.681 ± 0.018 | 0.431 ± 0.031 | 0.101 ± 0.005 | 0.071 ± 0.005 |
| Walnut | DBH | Western Indiana | 3–20 | 0.681 ± 0.018 | 0.501 ± 0.026 | 0.130 ± 0.012 | 0.095 ± 0.010 |
| Walnut | Height | Michigan | 3–10 | 0.854 ± 0.066 | 0.123 ± 0.013 | 0.207 ± 0.009 | 0.119 ± 0.012 |
| Walnut | DBH | Michigan | 5–10 | 1.752 ± 0.179 | 0.211 ± 0.079 | 0.364 ± 0.020 | 0.255 ± 0.022 |
| Walnut | Height | Southern Indiana | 3–15 | 0.938 ± 0.027 | 0.639 ± 0.025 | 0.174 ± 0.004 | 0.152 ± 0.004 |
| Walnut | DBH | Southern Indiana | 5–15 | 1.354 ± 0.063 | 0.803 ± 0.057 | 0.157 ± 0.007 | 0.118 ± 0.008 |

**Table S1** summarizes height- and DBH-based growth metrics for northern red oak and black walnut across high- and low-performance blocks. Across most plots and measurement types, high-performance blocks exhibited greater AGR and RGR values than low-performance blocks, indicating enhanced growth under favorable site conditions. Differences between performance classes were generally more pronounced for DBH than height measurements, particularly in walnut plots. Because measurement intervals varied among plots, comparisons are intended primarily within plots rather than across sites. These supplemental data provide interval-specific context supporting the broader growth patterns described in the main manuscript.

**Table S2.** Mean (± SEM) tree height and DBH at the beginning and end of each analyzed measurement interval across locations and treatments.

| **Plot Location** | **Species** | **Perf.** | **Interval** | **Year** | **Year** | **Height (m)** | **Height (m)** | **Dbh (cm)** | **Dbh (cm)** |
| --- | --- | --- | --- | --- | --- | --- | --- | --- | --- |
|  |  |  | **(yr)** | **start** | **end** | **Start (mean ± SEM)** | **End (mean ± SEM)** | **Start (mean ± SEM)** | **End (mean ± SEM)** |
| Western Indiana | Oak | Low | 5–15 | 5 | 15 | 2.603 ± 0.278 | 8.821 ± 0.468 | 1.887 ± 0.327 | 7.751 ± 0.800 |
| Western Indiana | Oak | High | 5–15 | 5 | 15 | 4.510 ± 0.191 | 13.193 ± 0.224 | 4.675 ± 0.218 | 18.493 ± 0.302 |
| Southern Indiana | Oak | Low | 5–15 | 5 | 15 | 2.220 ± 0.110 | 7.005 ± 0.214 | 1.750 ± 0.520 | 5.281 ± 0.339 |
| Southern Indiana | Oak | High | 5–15 | 5 | 15 | 3.927 ± 0.231 | 12.706 ± 0.135 | 3.275 ± 0.210 | 17.875 ± 0.545 |
| Michigan | Oak | Low | 3–15 | 3 | 15 | 1.443 ± 0.138 | 3.202 ± 0.284 | 0.731 ± 0.157 | 2.086 ± 0.246 |
| Michigan | Oak | High | 3–15 | 3 | 15 | 1.773 ± 0.210 | 11.531 ± 0.372 | 0.790 ± 0.119 | 14.750 ± 0.633 |
| Western Indiana | Walnut | Low | 3–20 | 3 | 20 | 3.153 ± 0.241 | 10.485 ± 0.525 | 2.450 ± 0.545 | 10.975 ± 0.778 |
| Western Indiana | Walnut | High | 3–20 | 3 | 20 | 2.586 ± 0.212 | 14.168 ± 0.115 | 2.729 ± 0.458 | 23.300 ± 0.948 |
| Southern Indiana | Walnut | Low | 3–15 | 3 | 15 | 1.480 ± 0.078 | 9.148 ± 0.318 | 2.687 ± 0.304 | 10.718 ± 0.709 |
| Southern Indiana | Walnut | High | 3–15 | 3 | 15 | 1.603 ± 0.090 | 12.858 ± 0.351 | 3.575 ± 0.195 | 17.118 ± 0.557 |
| Michigan | Walnut | Low | 3–10 | 3 | 10 | 0.674 ± 0.063 | 1.537 ± 0.115 | 0.487 ± 0.265 | 1.542 ± 0.655 |
| Michigan | Walnut | High | 3–10 | 3 | 10 | 1.886 ± 0.180 | 7.865 ± 0.591 | 1.770 ± 0.222 | 10.530 ± 1.002 |

**Table S2** provides the mean (± SEM) tree height and diameter at breast height (DBH) values measured at the beginning and end of each interval used for growth analyses across species, locations, and treatment groups. These data are included as supplemental material to provide the underlying measurements supporting growth patterns and interpretations described in the main manuscript. Intervals correspond to those used for absolute growth rate (AGR) and relative growth rate (RGR) calculations and therefore vary among species, trait, and site combinations according to available measurement years and analytical inclusion criteria. Reporting the start and end measurements allows readers to directly evaluate the observed changes underlying interval-based growth metrics. Height values are reported in meters (m) and DBH values in centimeters (cm).

**Table S3**: Pairwise PERMANOVA analysis based on location.

|  | pairs | Df | SumsOfSqs | F.Model | R2 | p.value |
| --- | --- | --- | --- | --- | --- | --- |
| 1 | West Indiana vs South Indiana | 1 | 0.9506103 | 2.90085 | 0.04611782 | 0.001 |
| 2 | West Indiana vs Michigan | 1 | 1.8027777 | 5.507689 | 0.08407699 | 0.001 |
| 3 | South Indiana vs Michigan | 1 | 2.3033238 | 6.902852 | 0.09735648 | 0.001 |

**Table S4**: Out-of-bag (OOB) error calculated for chemical parameters and recalculated with microbial taxa.

| Plant Species | Paramenters | Only chemical | Chemical+identified taxa |
| --- | --- | --- | --- |
| *J. nigra* | Dbh | 37.42 | 32.68 |
|  | Height | 8.94 | 8.6 |
| *Q. rubra* | Dbh | 29.16 | 30.67 |
|  | Height | 9.72 | 8.82 |

**Fig. S1:** Record of **A)** overall average annual high and low temperature and **B)** precipitation for each study site from the time of initial planting

**Fig. S2:** Record of *Quercus* *rubra* **A)** Na and K, **B)** inorganic carbon, pH (CaCl2 and H2O), Ca, and Mg, **C)** cation exchange capacity (base saturation), and **D)** CEC (NH4OAc-extracted), Bray 1P, %Clay, %Silt, %Sand, and A layer depth. Error bars ±SEM.

**Fig. S3:** Record of *Juglans nigra* **A)** Na and K, **B)** inorganic carbon, pH (CaCl2 and H2O), Ca, and Mg, **C)** cation exchange capacity (base saturation), and **D)** CEC (NH4OAc-extracted), Bray 1P, %Clay, %Silt, %Sand, and A layer depth. Error bars ±SEM.

**Fig. S4:** Recorded average height and DBH for the *Quercus rubra* and *Juglans nigra* planting sites. Error bars ±SEM.

**Fig. S5:** Dot plot showing the PCoA including the DNA control water and the soil samples.

**Fig. S6:** Pearson correlation between A, C) diameter of breast height (DBH) and B, D) tree height with A, B) sand content and C, D) pH for the three sites, both plant species ( *J. nigra* and *Q. rubra* ) and the plant development state (healthy and poor).

**Fig. S7:** Random forest mean predictor importance (% of increase in mean square error) of soil properties and diameter of breast height (DBH) for A) *J. nigra* and B) *Q. rubra*. This accuracy importance measure was computed for each tree and averaged over the forest (5000 trees). (n = 8).

**Fig. S8:** Pearson correlation between A) tree height and B) diameter of breast height (DBH) with the alpha-diversity index, measured as Shannon index, for the three sites, both plant species ( *J. nigra* and *Q. rubra* ) and the plant development state (healthy and poor).

**Fig. S9:** Mantel test correlation between soil bacterial structure, measured as axis 1 of the Bray-Curtis dissimilarity distance at the bacterial ASV level, and the soil characteristic, measured as axis 1 of the Bray-Curtis dissimilarity distance for the soil parameters, for A) *J. nigra* and B) *Q. rubra.*

**Fig. S10:** Dot plot showing the Spearman correlation between diameter of breast height (DBH) and A) *Bacillus*, B) *Gp3*, C) *Gp1*, D) *Aciditerrimonas*, E) *Arthrobacter*, F) *Chitinophagaceae_unclassified* for the three sites, both plant species ( *J. nigra* and *Q. rubra* ) and the plant development state (healthy and poor).
